# Differential DNA Methylation and Gene Expression Between ALV-J-Positive and ALV-J-Negative Chickens

**DOI:** 10.3389/fvets.2021.659840

**Published:** 2021-05-31

**Authors:** Yiming Yan, Huihua Zhang, Shuang Gao, Huanmin Zhang, Xinheng Zhang, Weiguo Chen, Wencheng Lin, Qingmei Xie

**Affiliations:** ^1^Guangdong Provincial Key Lab of AgroAnimal Genomics and Molecular Breeding, College of Animal Science, South China Agricultural University, Guangzhou, China; ^2^Key Laboratory of Animal Health Aquaculture and Environmental Control, Guangzhou, China; ^3^South China Collaborative Innovation Center for Poultry Disease Control and Product Safety, Guangzhou, China; ^4^College of Life Science and Engineering, Foshan University, Foshan, China; ^5^United States Department of Agriculture (USDA), Agriculture Research Service, Avian Disease and Oncology Laboratory, East Lansing, MI, United States

**Keywords:** MeDIP-Seq, RNA-Seq, integrated analysis, TGFB2, ALV-J-positive chickens

## Abstract

**Background:** Avian leukosis virus subgroup J (ALV-J) is an oncogenic virus that causes serious economic losses in the poultry industry; unfortunately, there is no effective vaccine against ALV-J. DNA methylation plays a crucial role in several biological processes, and an increasing number of diseases have been proven to be related to alterations in DNA methylation. In this study, we screened ALV-J-positive and -negative chickens. Subsequently, we generated and provided the genome-wide gene expression and DNA methylation profiles by MeDIP-seq and RNA-seq of ALV-J-positive and -negative chicken samples; 8,304 differentially methylated regions (DMRs) were identified by MeDIP-seq analysis (*p* ≤ 0.005) and 515 differentially expressed genes were identified by RNA-seq analysis (*p* ≤ 0.05). As a result of an integration analysis, we screened six candidate genes to identify ALV-J-negative chickens that possessed differential methylation in the promoter region. Furthermore, TGFB2 played an important role in tumorigenesis and cancer progression, which suggested TGFB2 may be an indicator for identifying ALV-J infections.

## Introduction

As a member of the Alpharetrovirus genus, avian leukosis virus (ALV) causes different pathotypes of neoplastic diseases in chickens (1, 2). The spread of ALVs leads to chicken slow growing, production performance degradation and caused serious losses to the poultry industry (3). According to their host range and viral envelope protein, ALVs can be classified as ALV-A, -B, -C, -D, -E, -J, and -K subgroups (4, 5). ALV-J was first isolated from meat-type chickens in England in 1988 (4). Since then, ALV-J has been the prevalent subtype of ALVs and has become a serious threat to the world's poultry industry (6, 7). To date, there is no effective vaccine against ALV-J. DNA methylation is a major epigenetic mechanism in eukaryotes and plays a crucial role in several biological processes, including the regulation of gene expression, embryonic development, X chromosome inactivation, and the development of various diseases (8–12). In mammals, DNA methylation occurs mainly at CpG islands and is generally associated with gene repression. However, aberrant methylation has been reported to be associated with various diseases, including neoplastic diseases. A number of studies have indicated that many tumor suppressor genes, such as FHIT, PTEN, and CMTM3, were silenced by promoter hypermethylation in the development of lung cancer, gynecological cancer, and gastric cancer (13–15). In general, DNA methylation maintains a stable state without environmental stimuli, but many factors can also change the DNA methylation patterns in organisms, including senility, and the development of diet and virus infection (16, 17).

The chicken (*Gallus gallus*) is considered to be an important animal model and, in 2011, the global DNA methylation patterns in chicken genome were analyzed (18). With the development of techniques for sequencing, more and more studies have been designed to identify the genome-wide methylation profiles of chickens, and the results have shown that DNA methylation plays a crucial role in chicken growth development, spermatogenesis, health status, and disease resistant (19–22). Recently, studies have focused on the interaction between a host response and pathogen infection. *Salmonella enterica* serovar Enteritidis inoculation has been shown to promote DNA methylation in chicken cecum, which implies that methylation of the HOX gene family may preform important regulatory functions in epigenetic regulation responding to SE inoculation in chickens (23). Marek's disease virus (MDV) can infect chickens causing neoplastic disease and can alter the genome-wide methylation levels of genes in the host (24). The hypomethylation of the CD30 gene occurs in all stages of tumorigenesis, and a high expression of CD30 could be a cue for lymphomas formation after MDV infection (63). ALV-J is also the virus associated with poultry tumors. In our previous study, we examined the aberrantly expressed microRNAs, mRNA, and circRNA in chickens challenged with ALV-J, some of which played important roles in tumorigenesis and the development of post ALV-J infection (25–28), although genome-wide DNA methylation variation of chickens infected with ALV-J was not fully clarified.

At present, the role of DNA methylation in the pathogenesis of diseases is the subject of intense investigation. In this study, we focused on comparing DNA methylation and gene expression between ALV-J-negative and -positive chickens by performing MeDIP-seq and RNA-seq analyses to identify the differences. Here, we provide basic data and explore the pathogenesis of ALV-J from the perspective of DNA methylation.

## Materials and Methods

### Ethics Statement

The animal study protocol was approved by the South China Agricultural University Committee of Animal Experiments (approval ID: SYXK-2019-0136). The experiments were closely followed in accordance with the recommendations of the Guide for the Care and Use of Laboratory Animals of the National Institutes of Health.

### Experimental Design

**Figure 1:**
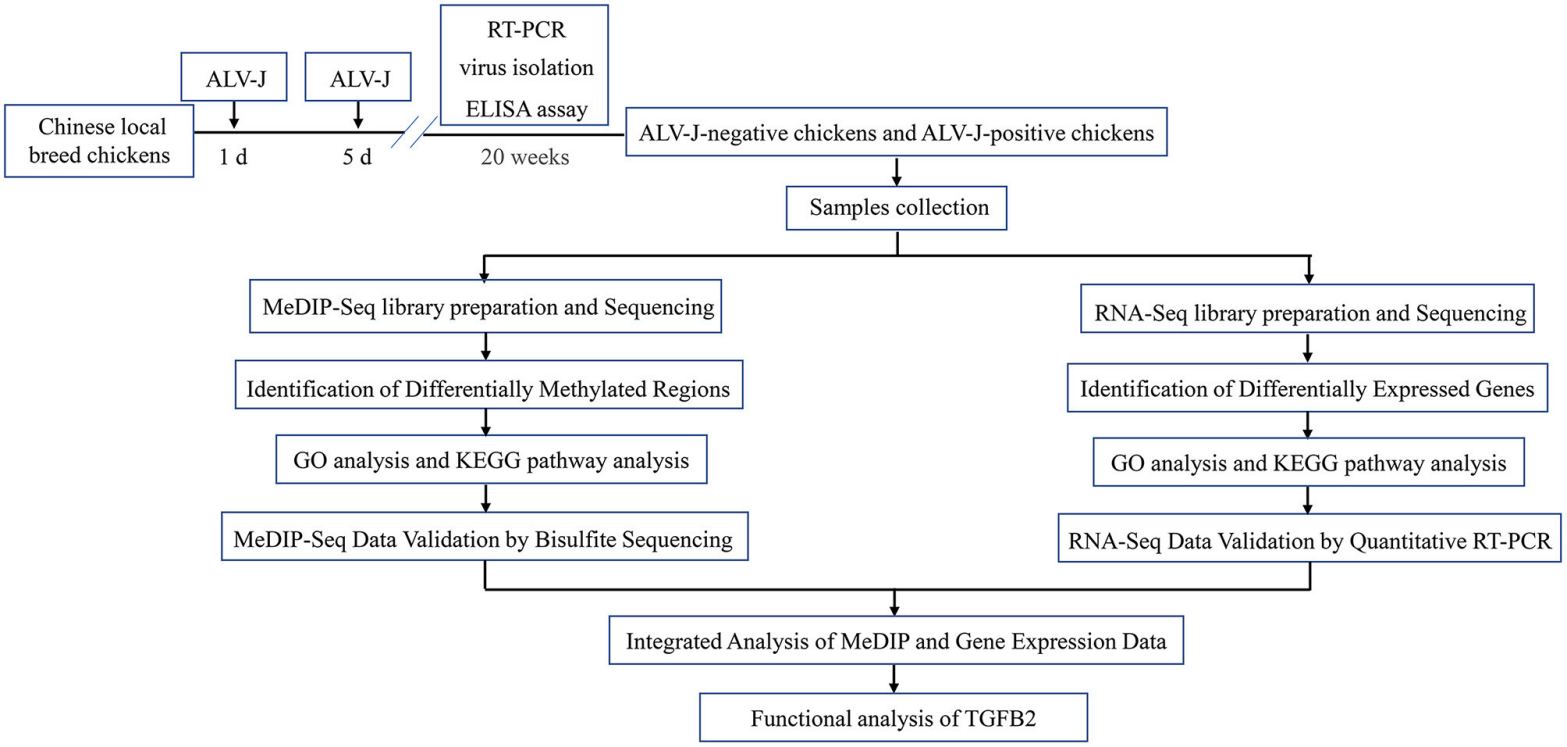
Experimental design schematization.

Experimental design is shown in Figure 1. The Chinese local breed chickens were used in this study. All of the 1-day-old chickens were intraperitoneally inoculated with ALV-J NX0101 at a dose of 0.2 mL (10^4.5^ TCID_50_/0.1 mL), and inoculated once again at 5 days old. The chickens were raised in negatively pressured biosecurity isolators under quarantine conditions and provided water and feed *ad libitum*. Whole blood and anti-coagulant blood samples were collected to detect virus or antibodies against ALV-J using reverse transcription PCR (RT-PCR), virus isolation and ELISA assay (29–31). Twenty weeks later, chickens tested without ALV-J infection during the period were designated as ALV-J-negative chickens, and the other chickens were identified as ALV-J-positive chickens. The livers of three ALV-J-negative chickens and three ALV-J-positive chickens were selected for MeDIP-Seq and RNA-Seq.

### Sample Collection

Three ALV-J-negative chickens and three ALV-J-positive chickens were euthanized to collect the whole livers for further analysis. All the samples were labeled with an ID and transported on dry ice to the laboratory for sample processing and testing. The livers (0.2 g each) were homogenized in phosphate-buffered saline (PBS), and then were detected by Immunofluorescence assay (IFA). Briefly, ALV-J in livers was detected by IFA using the ALV-J-specific monoclonal antibody JE9. Frozen sections of the livers were homogenized, filtered, and then DF-1 cells were inoculated with liver homogenate in a 24-well plate, followed by incubation at 37°C and 5% CO_2_ for 5 days; the cells were incubated with JE9 and detected using FITC-labeled anti-mouse IgG (Biolegend, USA). The fluorescence signals were viewed under a fluorescence microscope (Nikon, Japan). The remaining portion was collected for MeDIP-Seq and RNA-Seq. After this study, the remaining chickens were released to the population for breed conservation.

### Preparation of the Sequencing Libraries and Sequencing

#### MeDIP-Seq Library Preparation and Sequencing

Genomic DNA from the samples was isolated using phenol–chloroform extraction, precipitated with ethanol, and sonicated to 100–500 bp using a Bioruptor (Diagenode), following the manufacture's protocol (cycle number 6 and cycle conditions (on/off time) 30/30). Sonicated DNA was end repaired, A-tailed, and ligated to adapters by using a NEBNext® Ultra™ DNA Library Prep Kit (NEB). Then, MeDIP-seq was performed with a monoclonal antibody against 5-methylcytosine (Active Motif), following the manufacturer's standard protocol. MeDIP DNA libraries were quantified using Quant-iT PicoGreen dsDNA Kits (Life Technologies) and subjected to high-throughput 150 base paired-end sequencing on an Illumina Hiseq sequencer (Cloud-seq Inc., Shanghai, China), according to the manufacturer's recommended protocol.

#### RNA-Seq Library Preparetion and Sequencing

First, total RNA (1 μg) was used for removing rRNAs using Ribo-Zero rRNA Removal Kits (Illumina, San Diego, CA, USA) following the manufacturer's instructions. Second, RNA libraries were constructed by using rRNA-depleted RNAs with TruSeq Stranded Total RNA Library Prep Kit (Illumina, San Diego, CA, USA) according to the manufacturer's instructions. Libraries were controlled for quality by detecting the library length distribution (Supplementary Figure 1), and quantified using the BioAnalyzer 2100 system (Agilent Technologies, Inc., USA). Lastly, 10 pM libraries were denatured as single-stranded DNA molecules, captured on Illumina flow cells, amplified *in situ* as clusters, and sequenced for 150 cycles on Illumina HiSeq Sequencer according to the manufacturer's instructions (Cloud-seq Inc., Shanghai, China).

### Bioinformatic Analysis

#### Identification of Differentially Methylated Regions

High-quality MeDIP-seq reads were aligned against the *Gallus gallus* genome (UCSC galGal4) using Bowtie 2 software [v2.2.4, (32)] and only uniquely mapped reads were used for further analysis. Peak calling was performed with MACS software [v1.4.3, (33)]. The peaks in which the midpoint of peaks were located in the region from 2 KB upstream to 2 KB downstream of TSS were defined as promoter peaks; the peaks in which the midpoint of peaks were located in the region from 20 KB upstream to 2 KB upstream of TSS were defined as upstream peaks; the peaks in which the midpoint of peaks were located in the introns were defined as intron peaks; the peaks in which the midpoint of peaks were located in the exons were defined as exon peaks; and the peaks in which the midpoint of peaks were not located in the regions which had been mentioned above were defined as intergenic peaks. Differentially methylated regions (DMRs) were identified by diffReps software (negative binomial test) [v1.55.4, (34)]. The *p*-value of the filtering standard was 0.005, and a 2-fold change in the difference of read numbers was used as a criterion for the DMRs.

#### Identification of Differentially Expressed Genes

RNA-seq high-throughput sequencing and subsequent bioinformatics analysis were done by Cloud-Seq Biotech (Shanghai, China). Briefly, paired-end reads were harvested from the Illumina HiSeq 4000 sequencer, and quality-controlled by Q30. After 3′ adaptor trimming and removal of low-quality reads by cutadapt software (v1.9.3), the high-quality clean reads were aligned to the reference genome (UCSC galGal4) with hisat2 software (v2.0.4). Then, guided by the Ensembl gtf gene annotation file, cuffdiff software (35) was used to obtain the gene level FPKM as the expression profiles of mRNA, fold change and *p*-value were calculated based on FPKM, and differentially expressed mRNA (DEGs) were identified. Genes with a *p*-value ≤ 0.05 and a log2-transformed value smaller than −1 or greater than 1 were considered to be statistically significant DEGs.

#### Gene-Ontology (GO) Annotation and the Kyoto Encyclopedia of Genes and Genomes (KEGG) Pathway

To further investigate the biological processes and functions associated with differentially expressed genes, we performed GO and KEGG pathway analysis. Genes exhibiting fold change ≥2 and *p*-value ≤ 0.05 in different samples were analyzed for GO enrichments using clusterProfiler (36, 37), and KEGG pathway enrichments using the DAVID functional annotation tool (http://david.abcc.ncifcrf.gov/). To select the significant GO terms and pathways, a Fisher's exact test followed by the Benjamini–Hochberg (BH) multiple testing correction was performed to calculate the threshold of significance.

### Validation of the Sequencing Data

#### MeDIP-Seq Data Validation by Bisulfite Sequencing

To verify differentially methylated genes between ALV-J-negative and -positive chickens, 1 ug of genomic DNA from ALV-J-negative and -positive chicken samples were treated with sodium bisulfite using an EpiTect Fast LyseAll Bisulfite Kit (QIAGEN, Germany). Primer sequences for the genes selected for validation are documented in Supplementary Table 1.

#### RNA-Seq Data Validation by Quantitative RT-PCR

A total of 500 ng RNA was used to synthesize cDNA using a PrimeScript RT Reagent Kit (Perfect Real Time, TaKaRa, Osaka, Japan). Quantitative real-time PCR was performed using the 2x SYBR Green qPCR Master Mix (Bimake, USA); qPCR cycling conditions were 95°C for 5 min, followed by 40 cycles of 95°C for 10 s, and 60°C for 30 s. Beta-actin was also amplified and used as a loading control. Relative gene expression was analyzed using the 2^−ΔΔCt^ method. The primer sets used for validation are documented in Supplementary Table 2.

### Integrated Analysis of MeDIP and Gene Expression Data

To identify epigenetically regulated genes, the MeDIP-seq and RNA-seq data were integrated. DEGs that retained DMRs in the regulatory regions were selected as candidate genes. Then, genes with upregulated methylated regions (FC ≥ 2, *p* ≤ 0.005) in the regulatory regions but downregulated expression levels (FC ≥ 2, *p* ≤ 0.05), or downregulated methylated regions (FC ≥ 2, *p* ≤ 0.005) in the regulatory regions but upregulated expression levels (FC ≥ 2, *p* ≤ 0.05) were selected as candidates. All analyses were based on galGal4.

### Western Blotting Detection for TGFB2 Protein

DF-1 cells were harvested (Sigma) and lysed in RIPA buffer. Proteins were separated by SDS-PAGE (12%), then transferred onto PVDF membranes (Millipore), and then detected using primary antibodies. The primary antibodies used were rabbit polyclonal anti-TGFB2 (1:1,000; Absin, Shanghai, China), anti-flag (1:1,000; CST, Boston, USA), and anti-beta-actin (1:1,000; CST, Boston, USA), which was used as a protein loading control. The secondary antibody was goat polyclonal anti-rabbit IgG (H+L)-horseradish peroxidase (HRP, Bioss Inc.). Western blot bands were quantified with Image-Pro Plus 6.0.

### Statistical Analysis

The date from the qPCR shown are mean ± SE from three independent experiments. GraphPad Prism (version 5) was used to process the data. Statistical significance was determined using Student's *t*-test, and *p* < 0.05 and *p* < 0.01 were considered to show significant differences between the groups.

## Results

### Identification of ALV-J-Negative and -Positive Chickens

**Figure 2:**
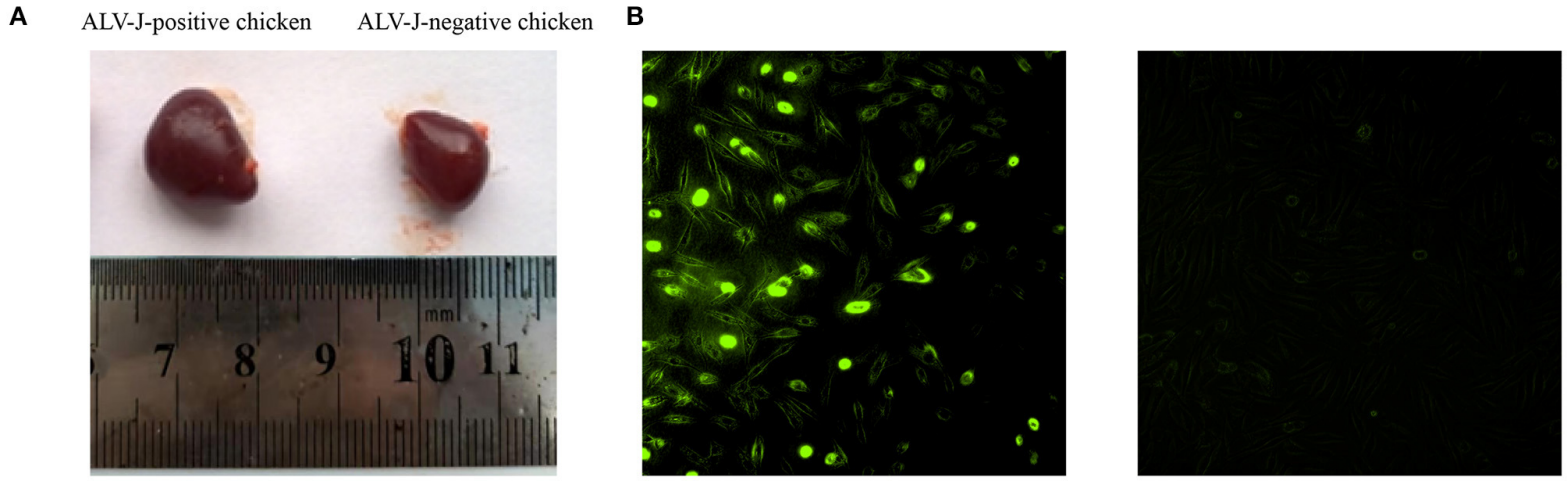
Characteristics of avian leukosis virus subgroup J (ALV-J)-negative and ALV-J-positive chicken samples. **(A)** Spleens of ALV-J-positive (left) and ALV-J-negative chickens (right); **(B)** immunofluorescence showed positive green fluorescence signals in DF-1 cells inoculated with liver homogenate of ALV-J-positive chickens (left) and no fluorescent signals in DF-1 cells inoculated with liver homogenate of ALV-J-negative chickens (right).

To screen the ALV-J-negative and -positive chickens, chickens were monitored for 20 weeks. At 20 weeks, we found that 10 chickens tested negative for ALV-J all the time, and 16 chickens were infected by ALV-J (Supplementary Table 3). Compared to the ALV-J-negative chickens, most of ALV-J-positive chickens showed gradual emaciation. The spleens of the ALV-J-positive chickens were significantly bigger than the ALV-J-negative chickens at 20 weeks (Figure 2A). DF-1 cells were inoculated with liver homogenate from six chickens and the livers of ALV-J-positive chickens were determined to be positive for p27 (Figure 2B). The results showed that we identified the ALV-J-negative chickens and ALV-J-positive chickens.

### Methylomic Profiling of ALV-J-Negative and -Positive Chickens

**Figure 3:**
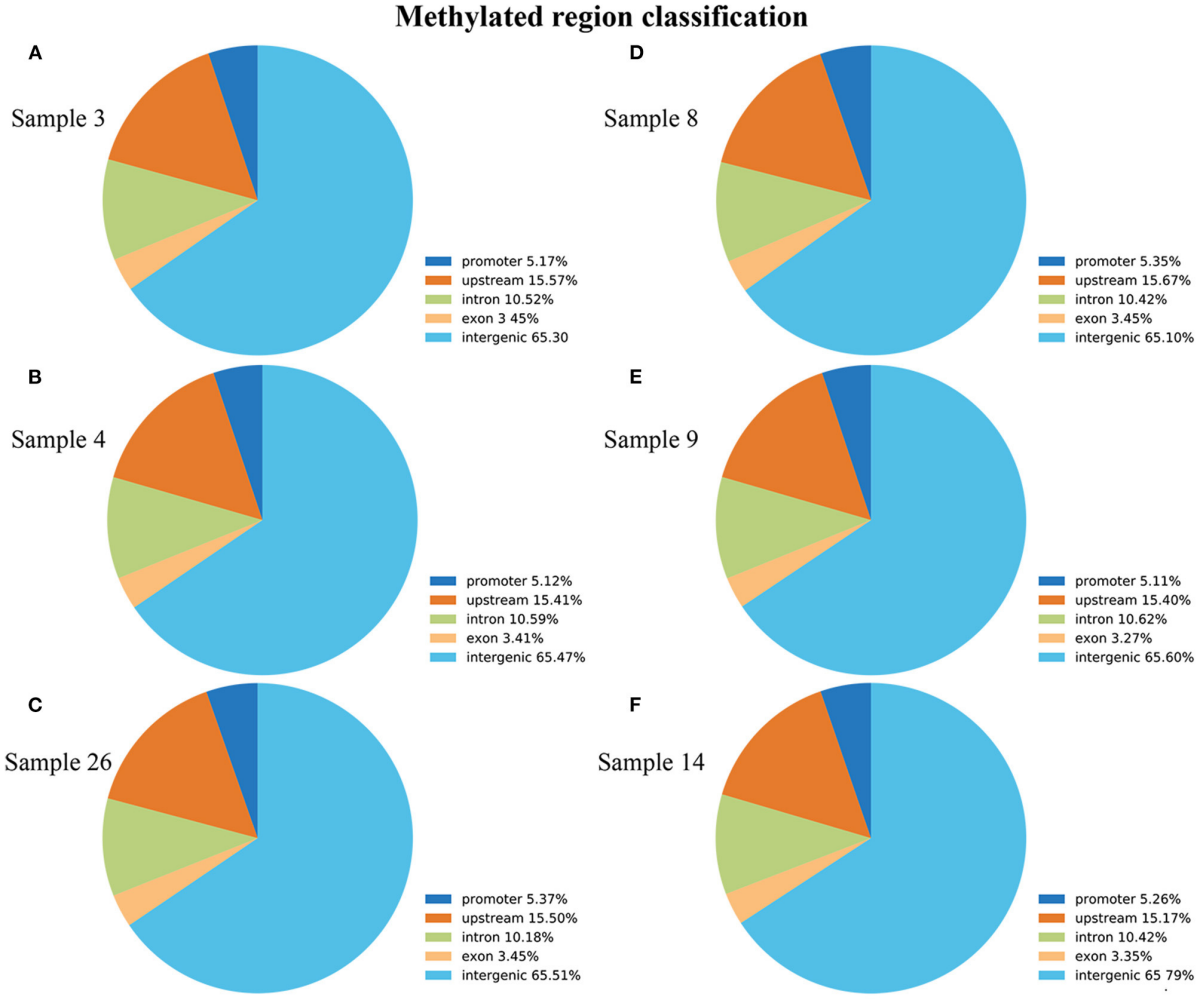
Distribution of methylation peaks in different genic regions of each sample, including promoter, intro, exon, intergenic, and upstream regions. **(A–C)** ALV-J-negative chickens; **(D–F)** ALV-J-positive chickens. Similar to the genic distribution of methylation peaks, the differentially methylated regions (DMRs) were most often located in the intergenic region in both groups.

To screen the methylomic profiling of ALV-J-negative and -positive chickens, the livers of the chickens were sampled, and MeDIP-seq was performed. Following the removal of low-quality data, we obtained a mean of 17,379,530 clean reads from the ALV-J-negative chicken samples and 17,674,372 clean reads from the ALV-J-positive chicken samples. The reads were mapped to the reference genome (UCSC galGal4), and mapping rates of 82.13–83.98% were obtained (Table 1). The reads were detected across all the mapped chicken chromosomes (Supplementary Figure 2). We analyzed the distribution of peaks among the different genomic components in each sample, including the promoter at the transcription start site, exon, intron, upstream, and the intergenic regions that contained the most peaks. The distribution of methylation peaks in different genomic regions showed similar pattern in those samples (Figure 3).

**Table 1:** Summary of reads generated by MeDIP-seq for per sample.

**Sample**	**Raw reads**	**Clean reads**	**Mapped to reference genome**	**Mapped percentage**
3	17,108,942	17,101,214	14,360,967	83.98%
4	17,719,218	17,708,194	14,779,696	83.46%
26	17,343,579	17,329,183	14,267,708	82.33%
8	16,993,746	16,982,842	14,048,795	82.72%
9	17,977,748	17,965,218	14,928,961	83.10%
14	18,087,379	18,075,056	14,844,676	82.13%

*Sample numbers 3, 4, and 26 represent ALV-J- negative chickens; 8, 9, and 14 represent ALV-J- positive chickens*.

### Characterization of Differential Methylated Regions and Validation of MeDIP-Seq Data Using Bisulfite Sequencing

**Figure 4:**
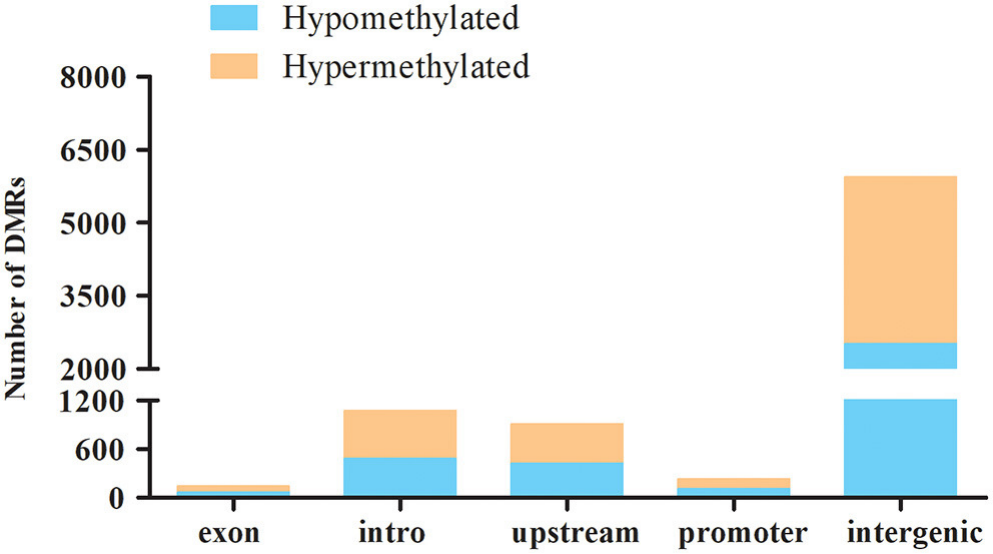
Distribution of the hyper- and hypomethylated DMRs in relation to the genes in the ALV-J-negative as compared with the -positive chicken samples. Distribution of hypermethylation and hypomethylation in each gene elements of ALV-J-positive chicken samples vs. ALV-J-negative chicken samples.

Differential methylation region (DMR) is considered to be a functional region regulating gene transcriptional level (38). DiffReps software was used to analyze DMRs in ALV-J-negative and -positive chicken samples. In total, 8,304 DMRs were identified between ALV-J positive and negative chickens. Being that 56.7% of them were hypermethylated and 43.3% were hypomethylated in the ALV-J-positive chickens (Supplementary Table 4). The DMR distribution showed that uniquely mapped reads in intergenic regions had a relatively higher methylation level than that of other regions, such as promoter, upstream, intron, and exon regions (Figure 4).

**Figure 5:**
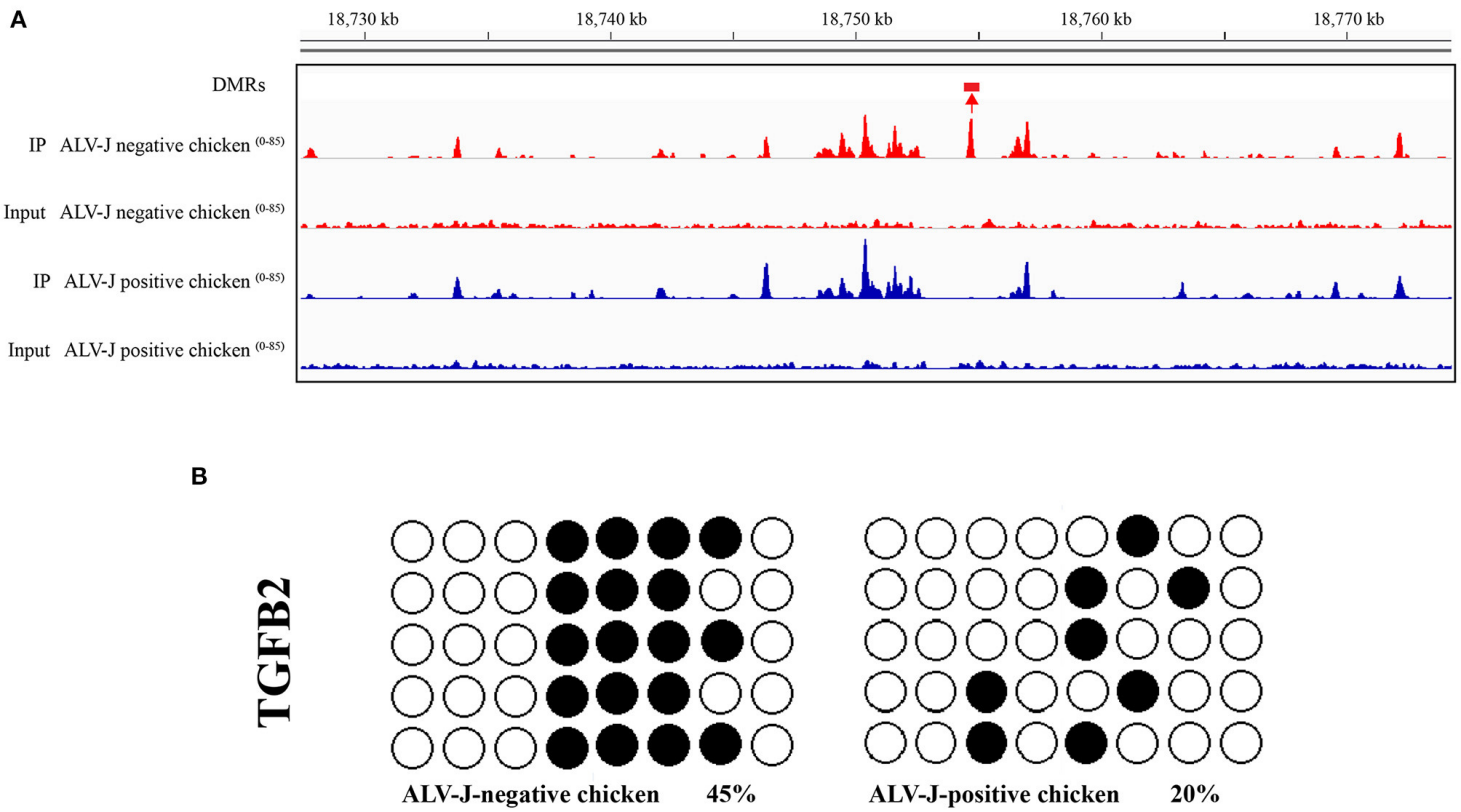
Validation of the hypomethylated gene TGFB2 in ALV-J- positive chicken samples vs. ALV-J- negative chicken samples by bisulfite sequencing. **(A)** Visualization of the DMRs in TGFB2, using the IGV tool. DMR is indicated by boxes above the tracks. Scales are showed on the left; **(B)** the validation result of the DMRs of TGFB2 in the promoter region by bisulfate sequencing. Black circles indicate the methylated CpG locus and white circles indicate the unmethylated CpG locus. The chi-square test showed the differences between ALV-J-negative and ALV-J-positive chickens.

To validate the MeDIP-seq data, we mainly focused on the analysis of DMRs located in the promoter region, which may be related to gene expression, therefore, we detected the methylation level of the TGFB2 gene. The bisulfite sequencing PCR (BSP) results confirmed the data accuracy of the MeDIP-seq analysis (Figures 5A,B).

**Figure 6:**
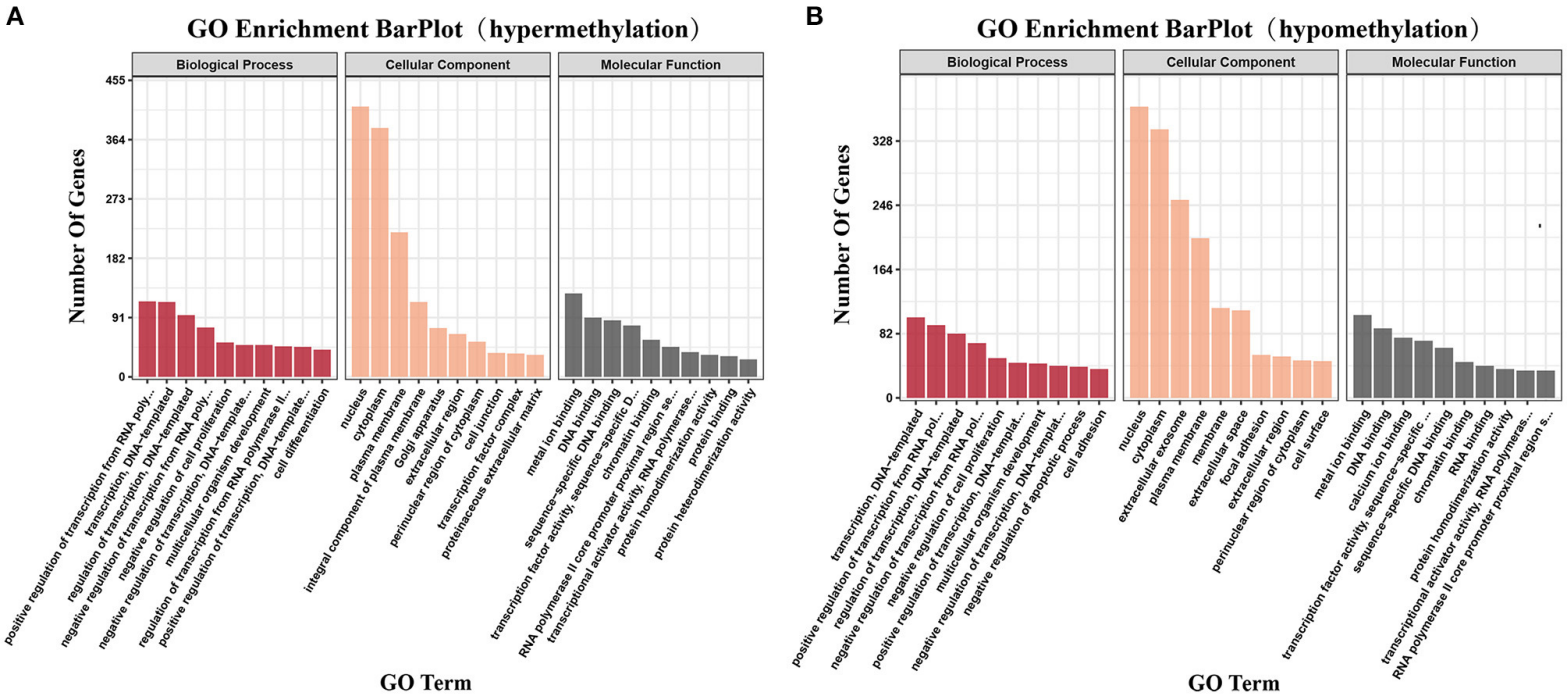
Gene ontology and pathway analysis of differentially methylated genes. **(A)** The significant gene ontology (GO) categories of hypermethylated DMRs in ALV-J-positive chickens as compared with ALV-J-negative chickens (*P* ≤ 0.05); **(B)** the significant GO categories of hypomethylated DMRs in ALV-J-positive chickens as compared with ALV-J-negative chickens (*P* ≤ 0.05).

We identified genes that contained DMRs among the methylated genes in ALV-J-negative and -positive chicken samples and GO analysis was carried out using DAVID software. GO terms with *p*-value ≤ 0.05 were considered to be functionally relevant. The GO analysis showed that 403 terms were enriched, including metal ion binding, DNA binding, and transcription, DNA-templated (Supplementary Table 5, Figure 6). The following four pathways were also identified: steroid biosynthesis, autophagy regulation, beta-alanine metabolism, and ABC transporters (*p* ≤ 0.05, Supplementary Table 6).

### Mapping of RNA-Seq Library Sequencing Data

**Figure 7:**
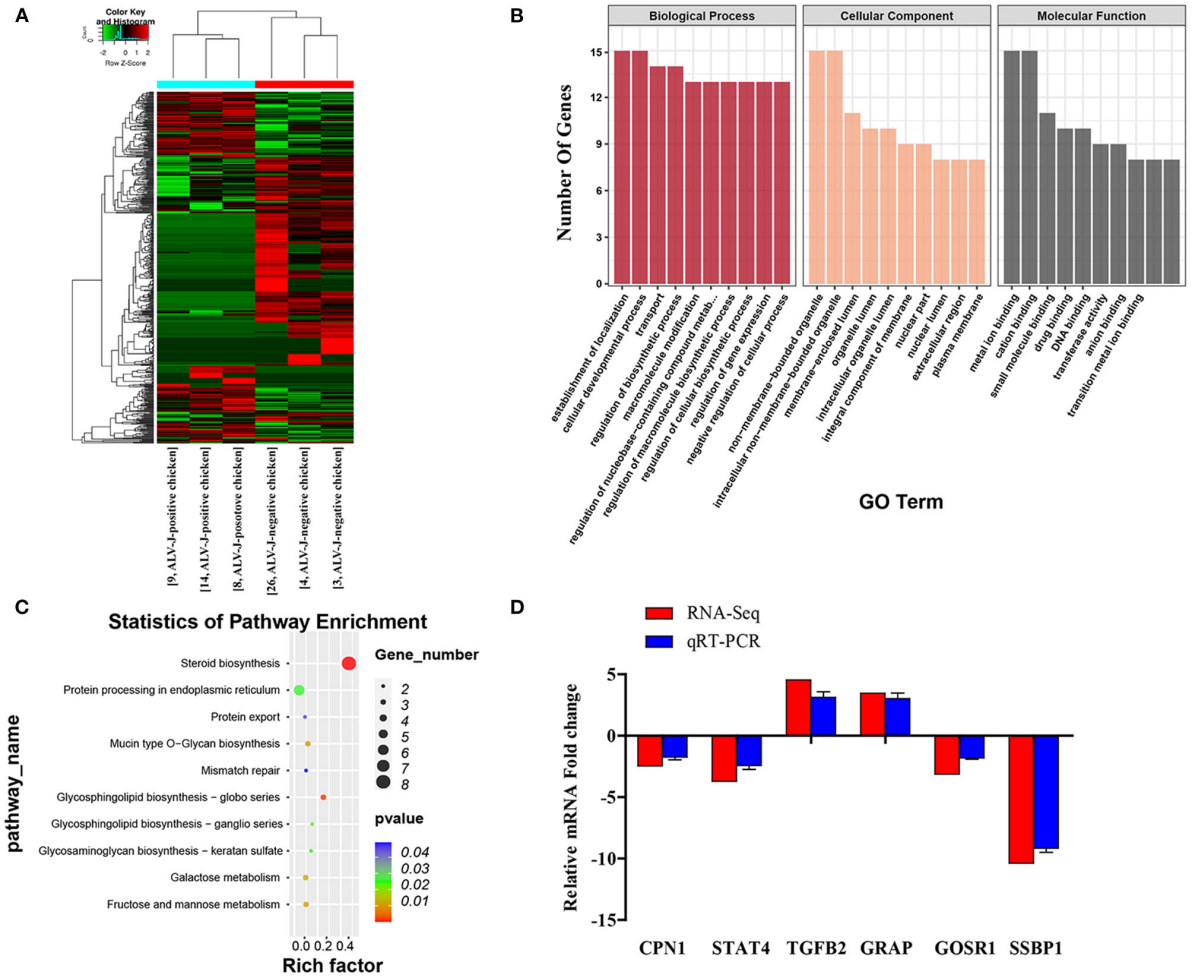
General profiling of the differentially expressed genes (DEGs). **(A)** Heatmap of DEGs from RNA-seq profiles; **(B)** the significant GO categories of the DEGs; **(C)** pathway analysis of the DEGs; **(D)** validation of differentially expressed genes by qRT-PCR. The vertical axis represents fold change of the RNA-seq and qRT-PCR in the ALV-J-positive chickens as compared with the ALV-J-negative chickens.

To identify mRNAs involved in disease resistance, total RNAs from ALV-J-negative and -positive chicken samples were used to construct small RNA libraries. From 22,442,240 to 31,752,754 clean reads were generated from three ALV-J-negative chicken samples and from 19,975,018 to 32,350,232 clean reads were generated from three ALV-J-positive chicken samples (Table 2). In total, there were 515 differentially expressed mRNAs identified, including 189 upregulated genes and 326 downregulated genes in ALV-J-positive chicken samples as compared with in ALV-J-negative chicken samples (Supplementary Table 7). The heatmap of all samples are shown in Figure 7A. To further understand the function of differentially expressed genes, GO and KEGG pathway analyses were conducted. Most of the GO terms were closely related to the establishment of localization and the cellular development process (Figure 7B and Supplementary Table 8). The KEGG pathway analysis showed that these DEGs were significantly involved in the glycosphingolipid biosynthesis globo series, steroid biosynthesis, and fructose and mannose metabolism pathways (Figure 7C and Supplementary Table 9). To validate the reliability of the RNA-seq data, qRT-PCR assays were performed. The results showed that qRT-PCR data for six mRNAs were consistent with the observed tendencies using RNA-seq (Figure 7D).

**Table 2:** Summary of reads generated by RNA-seq for each sample.

**Sample**	**Raw reads**	**Clean reads**	**Overall aligned reads**	**Overall alignment rate**
3	31,972,032	31,752,754	27,232,317	85.76%
4	29,101,802	28,323,384	25,108,913	88.65%
26	23,346,692	22,442,240	17,732,522	79.01%
8	28,812,098	27,209,832	21,958,483	80.70%
9	22,150,968	19,975,018	16,079,228	80.50%
14	33,766,962	32,350,232	24,801,434	76.67%

*Sample numbers 3, 4, and 26 represent ALV-J- negative chickens; 8, 9, and 14 represent ALV-J- positive chickens*.

### Integrated Analysis of MeDIP-Seq and RNA-Seq

**Figure 8:**
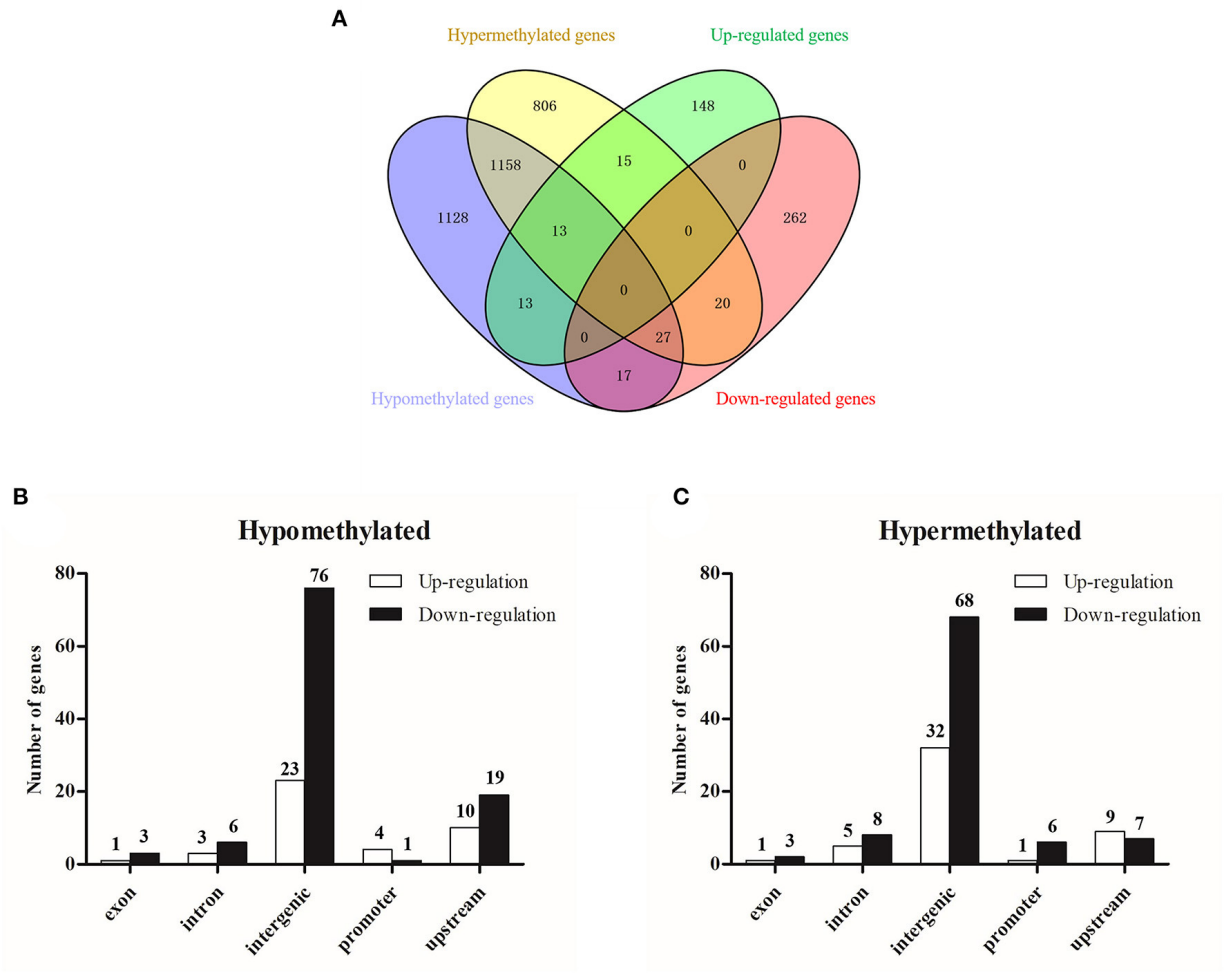
Integrated analysis of MeDIP-seq and RNA-seq data. **(A)** Venn diagram of differentially methylated genes and differentially expressed genes; **(B)** differentially hypermethylated genes with various genomic elements; **(C)** differentially hypomethylated genes with various genomic elements.

The integrated analysis between methylation and transcriptome was based on the data from MeDIP-seq and RNA-seq (Supplementary Table 10). The comprehensive distribution patterns of the genes with both differential methylation and expression levels are illustrated in Figure 8A. After merging overlapping DMGs containing DMRs with different gene elements into the unitary DMG, a total of 3,197 DMGs were identified. The genes located in various genomic regions are shown in Figures 8B,C. Furthermore, we observed 72 DEGs that might be regulated by aberrant DNA methylation, including 44 hypermethylation-low expressed genes and 28 hypomethylation-high expressed genes, which were considered to be potential candidate genes for ALV-J infection. In particular, TMEM104, FAM173A, CNP1, HSD17B7, SUPV3L1, and TGFB2 exhibited differential methylation in the promoter region (Table 3). Furthermore, we also analyzed six of the abovementioned genes with *Gallus gallus* genome version 6, and the results were similar with those when we used *Gallus gallus* genome version 4 (Supplementary Table 11).

**Table 3:** Differentially methylated and regulated genes with methylation profiles in promoter regions.

**GeneID**	**Chromosome**	**Genomic context**	**Methylated region**	**Fold change (MeDIP-seq)**	***p*-value**	**Fold change (RNA-seq)**	***p*-value**
CNP1	chr1	promoter	76428141–76428600	2.462	2.28E-01	−5.171	2.35E-02
FAM173A	chr14	promoter	13177801–13178160	2.869	1.77E-01	−3.338	9.10E-03
TMEM104	chr18	promoter	10726761–10727020	4.796	2.14E-02	−4.743	5.00E-05
HSD17B7	chr8	promoter	3791861–3792360	−3.125	6.14E-06	2.799	3.16E-02
SUPV3L1	chr6	promoter	10421761–10422180	−2.601	1.89E-04	2.309	3.06E-02
TGFB2	chr3	promoter	18754421–18754940	−5.082	1.67E-11	4.540	5.00E-05

*The DNA methylation and gene expression statuses of the ALV-J- positive chicken samples were utilized as references for those in the ALV-J- negative chicken samples*.

### Bisulfite Sequencing and qPCR Analysis of TGFB2 Expression Level in Chickens

**Figure 9:**
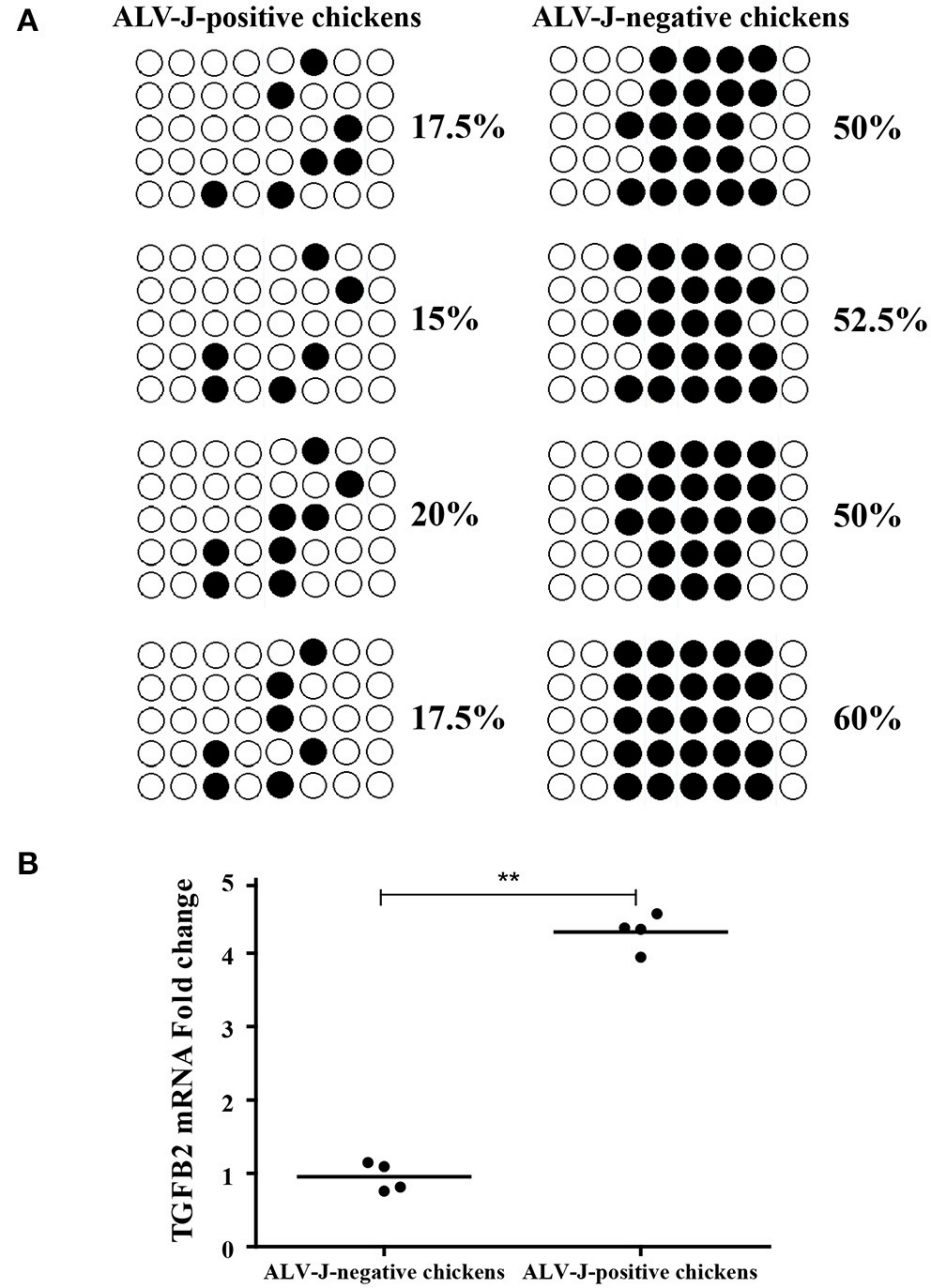
Identification of TGFB2 methylation level and expression in randomly selected chickens by bisulfite sequencing PCR (BSP) and qRT-PCR. **(A)** The methylation status of TGFB2 in promoter region was investigated among randomly selected chickens by BSP; **(B)** relative expression of TGFB2 in randomly selected chickens. Data represent means ± SE. **p < 0.01.

Furthermore, we analyzed the DNA methylation level of TGFB2 in another four randomly selected ALV-J-negative and four ALV-J-positive chickens. As a result, the methylation levels of TGFB2 in ALV-J-negative chickens were higher than that in ALV-J-positive chickens, essentially those results were consistent with MeDIP-seq data (Figure 9A). In particular, there was a methylation site found only in ALV-J-negative chickens. At the same time, we identified whether TGFB2 was commonly downexpressed in ALV-J-negative chickens, the abovementioned chickens which had been analyzed by qRT-PCR. In general, the results were in line with the RNA-seq data (Figure 9B). Taken together, we found TGFB2 underwent a massive loss of DNA methylation in the promoter region and the expression of TGFB2 was increased during ALV-J infection.

### Effect of TGFB2 on ALV-J Replication in DF-1 Cells

**Figure 10:**
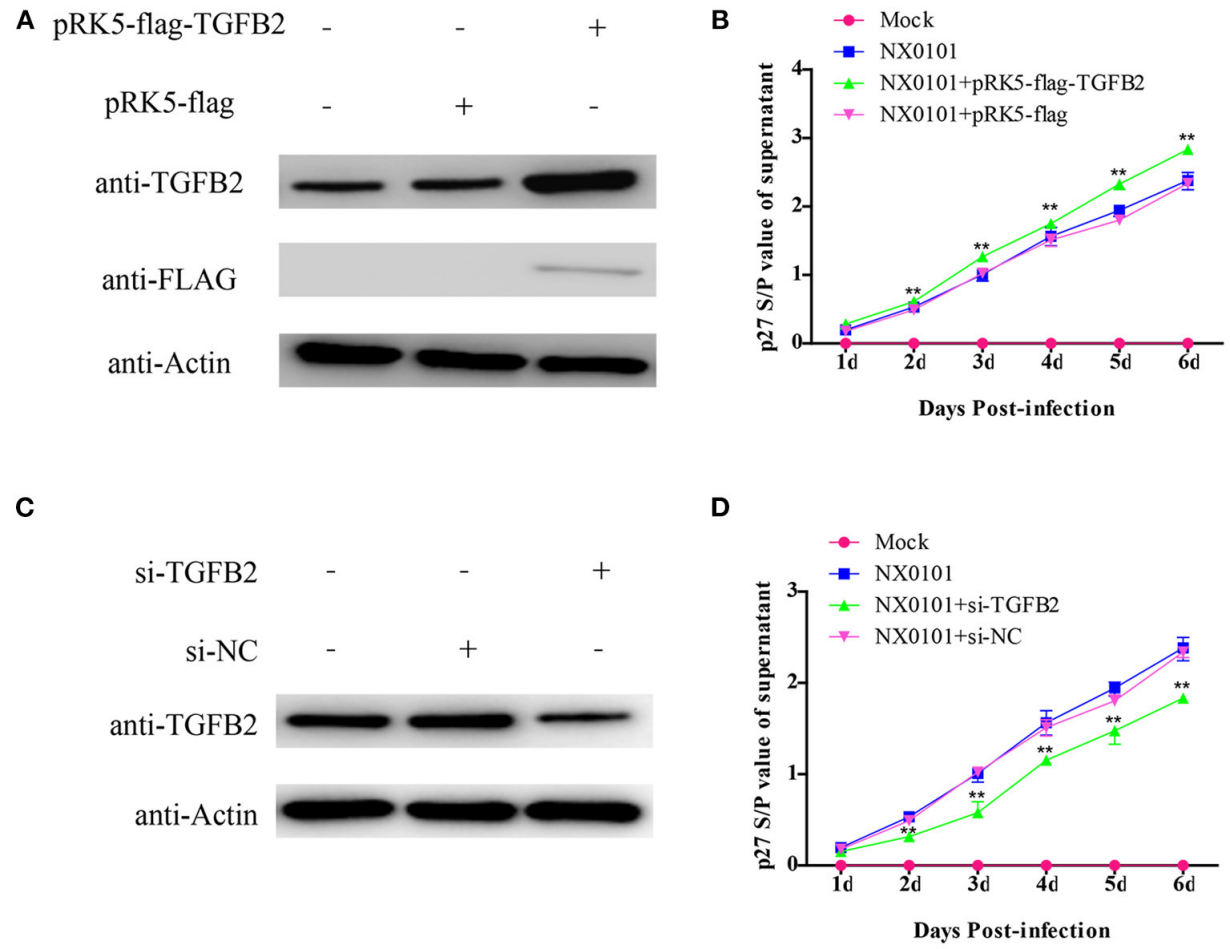
Effects of TGFB2 on ALV-J replication. **(A)** Expression levels of TGFB2 protein were analyzed by Western blotting 24 h after transfection of DF-1 cells with pRK5-flag-TGFB2 or pRK5-flag; **(B)** viral growth curve assay in DF-1 cells transfected with pRK5-flag-TGFB2 or pRK5-flag; **(C)** expression levels of TGFB2 protein were analyzed by Western blotting 24 h after transfection of DF-1 cells with siRNA targeted to TGFB2 or si-NC; **(D)** viral growth curve assay in DF-1 cells transfected with si-TGFB2 or si-NC. Data represent means ± SE. **p < 0.01.

In order to understand the reason for TGFB2 with different methylation levels and expression levels, we evaluated the function of TGFB2 in ALV-J replication. We transfected the TGFB2 expression vector into DF-1 cells. The replication of ALV-J was significantly promoted in the pRK5-flag-TGFB2-transfected group as compared with the pRK5-flag-transfected group (Figures 10A,B). Meanwhile, to identify whether low expression of TGFB2 would repress replication of ALV-J in DF-1 cells, we transfected DF-1 cells with si-RNA for TGFB2. The replication of ALV-J was significantly repressed in the DF-1 cells transfected with si-RNA for TGFB2, but not si-NC (Figures 10C,D). These results demonstrated the role of TGFB2 in ALV-J replication.

## Discussion

Tumor diseases are a serious problem in the poultry industry globally. It is known that ALV-J is associated with several kinds of tumors, such as hemangiomas, erythroblastosis and myelocytomas (39). Vertical transmission of the virus could cause severe immunosuppression in progenies (40), posing significant challenges to the control of this disease. Thus, it is necessary to screen potential molecular markers to identify ALV-J-positive chickens. In this study, we collected liver tissues from ALV-J-positive and -negative chickens and analyzed the DNA methylation because of the tumor emergence in livers of ALV-J-positive chickens in the field.

DNA methylation is an essential inheritable modification in most eukaryotic genomes, which plays a crucial role in regulating gene expression. However, this modification is usually altered in tumor cells. The alterations in DNA methylation patterns always affect the susceptibility of cancer. Although several reports regarding the effect of DNA methylation of a gene body on gene expression are available (41, 42), the function of DNA methylation of a gene body remains controversial. In contrast, the regulatory effects of DNA methylation of promoter regions on gene expression have been extensively proven in the past years. Estrogen receptor alpha (ERα) which plays an important role in controlling sexual development, is regulated by DNA methylation of promoter regions (43). A change in early growth response 1 (EGR1) expression has been derived from the aberrant methylation of the EGR1 gene promoter region in schizophrenia, and increased ERG1 expression might be connected with the pathophysiology of schizophrenia (44). Concerning glycoprotein VI (GPVI), it has been found that demethylation in the promoter region increased the expression of GPVI, which may be related to the occurrence of coronary heart disease (45). In omental adipocytes, the methylation of CYPl19A1 promoter region has been shown to be negatively associated with relative mRNA expression (46). In patients with type 2 diabetes, DNA methylation of the insulin promoter was increased as compare with non-diabetic donors, and correlated negatively with insulin gene expression in human pancreatic islets (47). All these reports have indicated that the DNA methylation of the promoter regions can regulate gene expression. In this study, we screened over 8,304 DMRs between ALV-J-positive and -negative chickens, and among these, 233 DMRs in the promoter region. More effort is required to analyze the functions of these DMRs in the future.

DNA methylation in the promoter region of genes plays an important role in the intricate host–disease interaction network (48). Abnormal DNA methylation during Marek's disease progression has indicated an interaction between MDV and the host gene, and has also found that aberrant methylation of the DNMT gene promoter region may also be involved in a virus-induced transformation process (24). The methylation level of ATF5 has been reported to be different in poorly differentiated glioma, well-differentiated glioma, and normal tissues, which suggested that aberrant methylation of ATF5 is connected with the pathophysiology of glioma (49). The methylation levels of SLC26A4 are significantly higher in patients with presbycusis and the methylation at the SLC26A4 promoter can predict the risk of presbycusis (50). Methylation in the p53 promoter region may play a key role in carcinogenesis of epithelial ovarian cancer and has been used as a molecular marker for screening of ovarian cancer (51). SLC5A8 has been shown to be a tumor suppressor in lung tumor and silenced by promoter hypermethylation in the development of lung cancer (52). Several studies have reported that there are some genes with aberrant DNA methylation in promoter regions in endometriosis, such as HOXA10 (53) and ATM (54). Taken together, these reports reveal that DNA methylation is implicated in the development of disease. Until now, there have been few studies regarding the association of DNA methylation with ALV-J infection. We integrated DNA methylation and gene expression data and identified a number of genes which simultaneously changed DNA methylation and gene expression. The integrated analysis showed that the genes expression levels of 72 genes were significantly inversely correlated with DNA methylation level in the ALV-J-negative chickens vs. the ALV-J-positive chickens. These results contributed to the exploration of the potential mechanism of epigenetic studies on the host response to ALV-J infection.

Transforming growth factor β2 (TGFB2), one of the isoforms of TGF-β, has been reported to be associated with various neoplasms (55–57). TGFB2 can initiate an autocrine loop that promotes its own expression and enables oncogenic activity. In gliomas, the expression levels of TGFB2 are used to evaluate stages of tumor progression (58, 59). In ovarian cancer, TGFB2 is overexpressed and plays a key role in ovarian oncogenesis by regulation of an epithelial-to-mesenchymal transition (60). TGFB2 has been shown to be changed in different tumor stages, T categories, grades, and patients' survival states, and upregulated in patients with GC as compared with a normal control, and its expression could be affected by cg11976166 (61). In prostate cancer, a quantitative increase in promoter methylation levels of TGFβ2 are associated with PCa progression (62). In this study, ALV-J-negative chickens had a higher level of TGFB2 promoter methylation than ALV-J-positive chickens and the DNA methylation of the TGFB2 promoter had a certain impact on TGFB2 gene expression. In addition, TGFB2 played a significant role in ALV-J replication and was verified *in vitro*. It is known that ALV-J can cause tumors in chickens; therefore, we suggest that the TGFB2 gene could be a marker gene to identify ALV-J infection.

## Data Availability Statement

The datasets presented in this study can be found in online repositories. The names of the repository/repositories and accession number(s) can be found below:

https://www.ncbi.nlm.nih.gov/, GSE163135

https://www.ncbi.nlm.nih.gov/, GSE143336.

## Ethics Statement

The animal study was reviewed and approved by the South China Agricultural University Committee of Animal Experiments. Written informed consent was obtained from the owners for the participation of their animals in this study.

## Author Contributions

YY: conceptualization, methodology, formal analysis, investigation, writing—original draft, writing—review and editing, and visualization. HuiZ: formal analysis, data curation, and visualization. SG: formal analysis, investigation, and visualization. HuanZ: supervision and writing—original draft. XZ: resources. WC: investigation. WL: writing—review and editing and resources. QX: conceptualization, supervision, funding acquisition, and project administration. All authors contributed to the article and approved the submitted version.

## Conflict of Interest

The authors declare that the research was conducted in the absence of any commercial or financial relationships that could be construed as a potential conflict of interest.

## Supplementary Material

Supplementary Figure 1 Quality assessment of RNA-Seq library.

Supplementary Figure 2 Distribution of MeDIP-Seq reads on each chromosome in each sample.

Supplementary Table 1 Information regarding the primers used to validate the methylation state of target gene promoter region by BSP.

Supplementary Table 2 Primers for qRT-PCR validation.

Supplementary Table 3 Results of infection status of chickens.

Supplementary Table 4 Total observerd DMRs in ALV-J-positive chickens and ALV-J-negative chickens.

Supplementary Table 5 Significantly enriched GO categories for DMRs.

Supplementary Table 6 Significantly enriched pathways for DMRs.

Supplementary Table 7 Total observerd DEGs in ALV-J-positive chickens and ALV-J-negative chickens.

Supplementary Table 8 Significantly enriched GO categories for DEGs.

Supplementary Table 9 Significantly enriched pathways for DEGs.

Supplementary Table 10 Integrated analysis of MeDIP-seq and RNA-seq data.

Supplementary Table 11 The six genes were reanalyze based on *galGal6*.

## Abbreviations

ALV: Avian Leukosis Virus
ALV-J: Avian leukosis virus subgroup J
CNP1: C-type natriuretic peptide 1
DEGs: differentially expressed genes
DMRs: differentially methylated regions
EGR1: early growth response 1
Erα: Estrogen receptor alpha
FAM173A, family with sequence similarity 173: member A
GO: gene ontology
GPVI: glycoprotein VI
HSD17B7: hydroxysteroid 17-beta dehydrogenase 7
IFA: immunofluorescence assay
KEGG: Kyoto Encyclopedia of Genes and Genomes
MDV: Marek's disease virus
MeDIP-Seq: methylated DNA immunoprecipitation sequencing
qRT-PCR: quantitative real time Polymerase Chain Reaction
RNA-Seq: quantitative measurements of transcriptomes
SUPV3L1: Suv3 like RNA helicase
TGFB2: transforming growth factor beta 2
TMEM104: transmembrane protein 104.

## References

[1] Payne LN Venugopal K. Neoplastic diseases: Marek's disease, avian leukosis and reticuloendotheliosis. Rev Sci Tech. (2000) 19:544. 10.20506/rst.19.21226 10935279

[2] Nakamura S Ochiai K Ochi A Yabushita H Abe A Kishi S. Cardiac pathology and molecular epidemiology by avian leukosis viruses in Japan. PLos ONE. (2014) 9:e86546. 10.1371/journal.pone0086546 24466146 PMC3900567

[3] Payne LN Nair V. The long view: 40 years of avian leukosis research. Avian Pathol. (2012) 41:11–9. 10.1080/03079457.2011646237 22845317

[4] Payne LN Brown SR Bumstead N Howes K Frazier JA Thouless ME. A novel subgroup of exogenous avian leukosis virus in chickens. J Gen Virol. (1991) 72:801–7. 10.1099/0022-1317-72-4-801 1849967

[5] Payne LN Gillespie AM Howes K. Unsuitability of chicken sera for detection of exogenous ALV by the group-specific antigen ELISA. Vet Rec. (1993) 132:555–7. 10.1136/vr.132.22555 7687401

[6] Fadly AM Smith EJ. Isolation and some characteristics of a subgroup J-like avian leukosis virus associated with myeloid leukosis in meat-type chickens in the United States. Avian Dis. (1999) 43:391–400. 10.2307/1592636 10494407

[7] Du Y Cui Z Qin A. Subgroup J avian leukosis viruses in China. China Poult Sci. (1999) 3:1–4.

[8] Abhishek S Palamadai KS. Epidermal differentiation complex: a review on its epigenetic regulation and potential drug targets. Cell J. (2016) 18:1–6. 10.22074/cellj.2016.3980 27054112 PMC4819378

[9] Douvaras P Rusielewicz T Kim K Haines J Casaccia P Fossati V. Epigenetic modulation of human induced pluripotent stem cell differentiation to oligodendrocytes. Int J Mol Sci. (2016) 17:614. 10.3390/ijms17040614 27110779 PMC4849063

[10] Liu Y Giannopoulou EG Wen D Falciatori I Elemento O Allis CD. Epigenetic profiles signify cell fate plasticity in unipotent spermatogonial stem and progenitor cells. Nat Commun. (2016) 7:11275. 10.1038/ncomms11275 27117588 PMC4853422

[11] Dawson MA Kouzarides T. Cancer epigenetics: from mechanism to therapy. Cell. (2012) 150:12–27. 10.1016/j.cell.2012.06013 22770212

[12] Goeppert B Konermann C Schmidt CR Bogatyrova O Geiselhart L Ernst C. Global alterations of DNA methylation in cholangiocarcinoma target the Wnt signaling pathway. Hepatology. (2014) 59:544–54. 10.1002/hep26721 24002901

[13] Zochbauer-Muller S Fong KM Maitra A Lam S Geradts J Ashfaq R. 5' CpG island methylation of the FHIT gene is correlated with loss of gene expression in lung and breast cancer. Cancer Res. (2001) 61:3581–5. 11325823

[14] Yang HJ Liu VW Wang Y Tsang PC Ngan HY. Differential DNA methylation profiles in gynecological cancers and correlation with clinico-pathological data. BMC Cancer. (2006) 6:212. 10.1186/1471-2407-6-212 16928264 PMC1560388

[15] Wang Y Li J Cui Y Li T Ng KM Geng H. CMTM3, located at the critical tumor suppressor locus 16q22.1, is silenced by CpG methylation in carcinomas and inhibits tumor cell growth through inducing apoptosis. Cancer Res. (2009) 69:5194–201. 10.1158/0008-5472CAN-08-3694 19509237

[16] Jaenisch R Bird A. Epigenetic regulation of gene expression: how the genome integrates intrinsic and environmental signals. Nat Genet. (2003) 33:245–4. 10.1038/ng1089 12610534

[17] Luo J Yu Y Zhang H Tian F Chang S Cheng HH. Down-regulation of promoter methylation level of CD4 gene after MDV infection in MD-susceptible chicken line. BMC Proc. (2011) 5:S7. 10.1186/1753-6561-5-S4-S7 21645322 PMC3108237

[18] Li Q Li N Hu X Li J Du Z Chen L. Genome-wide mapping of DNA methylation in chicken. PLoS ONE. (2011) 6:e19428. 10.1371/journal.pone0019428 21573164 PMC3088676

[19] Hu Y Xu H Li Z Zheng X Jia X Nie Q. Comparison of the genome-wide DNA methylation profiles between fast-growing and slow-growing broilers. PLoS ONE. (2013) 8:e56411. 10.1371/journal.pone0056411 23441189 PMC3575439

[20] Li Q Wang Y Hu X Zhao Y Li N. Genome-wide mapping reveals conservation of promoter DNA methylation following chicken domestication. Sci Rep. (2015) 5:8748. 10.1038/srep08748 25735894 PMC4348661

[21] Raddatz G Arsenault RJ Aylward B Whelan R Bohl F Lyko F. A chicken DNA methylation clock for the prediction of broiler health. Commun Biol. (2021) 4:76. 10.1038/s42003-020-01608-7 33462334 PMC7814119

[22] Li Y Ding X Wang X He T Zhang H Yang L. Genome-wide comparative analysis of DNA methylation between soybean cytoplasmic male-sterile line NJCMS5A and its maintainer NJCMS5B. BMC Genomics. (2017) 18:596. 10.1186/s12864-017-3962-5 28806912 PMC5557475

[23] Wang Y Liu L Li M Lin L Su P Tang H. Chicken cecal DNA methylome alteration in the response to Salmonella enterica serovar Enteritidis inoculation. BMC Genomics. (2020) 21:814. 10.1186/s12864-020-07174-w 33225883 PMC7681971

[24] Luo J Yu Y Chang S Tian F Zhang H Song J. DNA methylation fluctuation induced by virus infection differs between MD-resistant and -susceptible chickens. Front Genet. (2012) 3:20. 10.3389/fgene.201200020 22363343 PMC3281210

[25] Li H Ji J Xie Q Shang H Zhang H Xin X. Aberrant expression of liver microRNA in chickens infected with subgroup J avian leukosis virus. Virus Res. (2012) 169:268–71. 10.1016/j.virusres.2012.07003 22800510

[26] Zhang X Yan Z Li X Lin W Dai Z Yan Y. GADD45β, an anti-tumor gene, inhibits avian leukosis virus subgroup J replication in chickens. Oncotarget. (2016) 7:68883–93. 10.18632/oncotarget12027 27655697 PMC5356597

[27] Zhang X Yan Y Lei X Li A Zhang H Dai Z. Circular RNA alterations are involved in resistance to avian leukosis virus subgroup-J-induced tumor formation in chickens. Oncotarget. (2017) 8:34961–70. 10.18632/oncotarget16442 28415618 PMC5471026

[28] Zhang X Yan Y Lin W Li A Zhang H Lei X. Circular RNA Vav3 sponges gga-miR-375 to promote epithelial-mesenchymal transition. RNA Biol. (2019) 16:118–32. 10.1080/15476286.20181564462 30608205 PMC6380338

[29] Li X Lin W Chang S Zhao P Zhang X Liu Y. Isolation, identification and evolution analysis of a novel subgroup of avian leukosis virus isolated from a local Chinese yellow broiler in South China. Arch Virol. (2016) 161:2717–25. 10.1007/s00705-016-2965-x 27422398

[30] Xie T Feng M Dai M Mo G Ruan Z Wang G. Cholesterol-25-hydroxylase is a chicken ISG that restricts ALV-J infection by producing 25-hydroxycholesterol. Viruses. (2019) 11:60498. 10.3390/v11060498 31151272 PMC6631237

[31] Bagust TJ Fenton SP Reddy MR. Detection of subgroup J avian leukosis virus infection in Australian meat-type chickens. Aust Vet J. (2004) 82:701–6. 10.1111/j.1751-0813.2004.tb12163x 15977617

[32] Langmead B Salzberg SL. Fast gapped-read alignment with Bowtie 2. Nat Methods. (2012) 9:357–9. 10.1038/nmeth1923 22388286 PMC3322381

[33] Zhang Y Liu T Meyer CA Eeckhoute J Johnson DS Bernstein BE. (2008). Model-based analysis of ChIP-Seq (MACS). Genome Biol. 9:R137. 10.1186/gb-2008-9-9-r137 PMC2592715 18798982

[34] Shen L Shao NY Liu X Maze I Feng J Nestler EJ. diffReps: detecting differential chromatin modification sites from ChIP-seq data with biological replicates. PLoS ONE. (2013) 8:e65598. 10.1371/journal.pone0065598 23762400 PMC3677880

[35] Trapnell C Williams BA Pertea G Mortazavi A Kwan G van Baren MJ. Transcript assembly and quantification by RNA-Seq reveals unannotated transcripts and isoform switching during cell differentiation. Nat Biotechnol. (2010) 28:511–5. 10.1038/nbt1621 20436464 PMC3146043

[36] Yu G Wang L Han Y He Q. clusterProfiler: an R Package for comparing biological themes among gene clusters. OMICS. (2012) 16:284–7. 10.1089/omi.20110118 22455463 PMC3339379

[37] Yu G Wang L Yan G He Q. DOSE: an R/Bioconductor package for disease ontology semantic and enrichment analysis. Bioinformatics. (2015) 31:608–9. 10.1093/bioinformatics/btu684 25677125

[38] Grimm C Chavez L Vilardell M Farrall AL Tierling S Bohm JW. (2013). DNAmethylome analysis of mouse intestinal adenoma identifies a tumour-specific signature that is partly conserved in human colon cancer. PLoS Genet. 9:e1003250. 10.1371/journal.pgen.1003250 PMC3567140 23408899

[39] Sironi G Manarolla G Pisoni G Recordati C Rampin T. Myotropic avian leukosis virus subgroup J infection in a chicken. J Vet Med B Infect Dis Vet Public Health. (2006) 53:347–9. 10.1111/j.1439-0450.2006.00961x 16930280

[40] Lin Y Xia J Zhao Y Wang F Yu S Zou N. Reproduction of hemangioma by infection with subgroup J avian leukosis virus: the vertical transmission is more hazardous than the horizontal way. Virol J. (2013) 10:97. 10.1186/1743-422X-10-97 23537218 PMC3717065

[41] Kulis M Heath S Bibikova M Queirós AC Navarro A Clot G. Epigenomic analysis detects widespread gene-body DNA hypomethylation in chronic lymphocytic leukemia. Nat Genet. (2012) 44:1236–42. 10.1038/ng2443 23064414

[42] Yang X Han H De Carvalho DD Lay FD Jones PA Liang G. Gene body methylation can alter gene expression and is a therapeutic target in cancer. Cancer Cell. (2014) 26:577–90. 10.1016/j.ccr.2014.07028 25263941 PMC4224113

[43] Westberry JM Trout AL Wilson ME. Epigenetic regulation of estrogen receptor alpha gene expression in the mouse cortex during early postnatal development. Endocrinology. (2010) 151:731–40. 10.1210/en2009-0955 19966177 PMC2817618

[44] Hu TM Chen SJ Hsu SH Cheng MC. Functional analyses and effect of DNA methylation on the EGR1 gene in patients with schizophrenia. Psychiatry Res. (2019) 275:276–82. 10.1016/j.psychres.2019.03044 30952071

[45] Gao S Han Y Chen X Dai L Gao H Lei Z. Epigenetic modulation of glycoprotein VI gene expression by DNA methylation. Life Sci. (2020) 241:117103. 10.1016/j.lfs.2019117103 31783053

[46] Lewis JR McNab TJ Liew LJ Tan J Hudson P Wang JZ. DNA methylation within the I.4 promoter region correlates with CYPl19A1 gene expression in human *ex vivo* mature omental and subcutaneous adipocytes. BMC Med Genet. (2013) 14:87. 10.1186/1471-2350-14-87 24128150 PMC3765767

[47] Yang BT Dayeh TA Kirkpatrick CL Taneera J Kumar R Groop L. Insulin promoter DNA methylation correlates negatively with insulin gene expression and positively with HbA(1c) levels in human pancreatic islets. Diabetologia. (2011) 54:360–7. 10.1007/s00125-010-1967-6 21104225 PMC3017313

[48] Zheng DL Zhang L Cheng N Xu X Deng Q Teng XM. Epigenetic modification induced by hepatitis B virus X protein via interaction with de novo DNA methyltransferase DNMT3A. J Hepatol. (2009) 50:377–87. 10.1016/j.jhep.2008.10019 19070387

[49] Hua XM Wang J Qian DM Song JY Chen H Zhu XL. DNA methylation level of promoter region of activating transcription factor 5 in glioma. J Zhejiang Univ Sci B. (2015) 16:757–62. 10.1631/jzusB1500067 26365117 PMC4569683

[50] Xu J Zheng J Shen W Ma L Zhao M Wang X. Elevated SLC26A4 gene promoter methylation is associated with the risk of presbycusis in men. Mol Med Rep. (2017) 16:347–52. 10.3892/mmr.20176565 28498466

[51] Chmelarova M Krepinska E Spacek J Laco J Beranek M Palicka V. Methylation in the p53 promoter in epithelial ovarian cancer. Clin Transl Oncol. (2013) 15:160–3. 10.1007/s12094-012-0894-z 22855178

[52] Park JY Kim D Yang M Park HY Lee SH Rincon M. Gene silencing of SLC5A8 identified by genome-wide methylation profiling in lung cancer. Lung Cancer. (2013) 79:198–204. 10.1016/j.lungcan.2012.11019 23273563 PMC3566332

[53] Wu Y Halverson G Basir Z Strawn E Yan P Guo SW. Aberrant methylation at HOXA10 may be responsible for its aberrant expression in the endometrium of patients with endometriosis. Am J Obstet Gynecol. (2005) 193:371–80. 10.1016/j.ajog.2005.01034 16098858

[54] Hirakawa T Nasu K Aoyagi Y Takebayashi K Zhu R Narahara H. ATM expression is attenuated by promoter hypermethylation in human ovarian endometriotic stromal cells. Mol Hum Reprod. (2019) 25:295–304. 10.1093/molehr/gaz016 30869775

[55] Joseph JV Conroy S Tomar T Eggens-Meijer E Bhat K Copray S. TGF-β is an inducer of ZEB1-dependent mesenchymal transdifferentiation in glioblastoma that is associated with tumor invasion. Cell Death Dis. (2014) 5:e1443. 10.1038/cddis.2014395 25275602 PMC4649508

[56] Niu G Li B Sun L An C. MicroRNA-153 inhibits osteosarcoma cells proliferation and invasion by targeting TGF-β2. PLoS ONE. (2015) 10:e0119225. 10.1371/journal.pone0119225 25793604 PMC4368543

[57] Lu R Ji Z Li X Qin J Cui G Chen J. Tumor suppressive microRNA-200a inhibits renal cell carcinoma development by directly targeting TGFB2. Tumor Biol. (2015) 36:6691–700. 10.1007/s13277-015-3355-9 25813153

[58] Jachimczak P Hessdorfer B Fabel-Schulte K Wismeth C Brysch W Schlingensiepen KH. Transforming growth factor-beta-mediated autocrine growth regulation of gliomas as detected with phosphorothioate antisense oligonucleotides. Int J Cancer. (1996) 65:332–7. 10.1002/(SICI)1097-0215(19960126)65:3 <332::AID-IJC10>3.0CO;2-C 8575854

[59] Kjellman C Olofsson SP Hansson O Von S.chantz T, Lindvall M, Nilsson I, et al. Expression of TGF-beta isoforms, TGF-beta receptors, and SMAD molecules at different stages of human glioma. Int J Cancer. (2000) 89:251–8. 10.1002/1097-0215(20000520)89:3 <251::AID-IJC7>3.0CO;2-5 10861501

[60] Do TV Kubba LA Du H Sturgis CD Woodruff TK. Transforming growth factor- 1, transforming growth factor- 2, and transforming growth factor- 3 enhance ovarian cancer metastatic potential by inducing a smad3-dependent epithelial-to-mesenchymal transition. Mol Cancer Res. (2008) 6:695–705. 10.1158/1541-7786MCR-07-0294 18505915 PMC2927222

[61] Li T Chen X Gu M Deng A Qian C. Identification of the subtypes of gastric cancer based on DNA methylation and the prediction of prognosis. Clin Epigenetics. (2020) 12:161. 10.1186/s13148-020-00940-3 33115518 PMC7592597

[62] Liu L Kron KJ Pethe VV Demetrashvili N Nesbitt ME Trachtenberg J. Association of tissue promoter methylation levels of APC, TGFbeta2, HOXD3 and RASSF1A with prostate cancer progression. Int J Cancer. (2011) 129:2454–62. 10.1002/ijc25908 21207416

[63] Li K Lian L Yang N Qu L. Temporal expression and DNA hypomethylation profile of CD30 in Marek's disease virus-infected chicken spleens. Poult Sci. (2015) 94:1165–9. 10.3382/ps/pev100 25840961

