# Supplementary figures and images for: Differential DNA Methylation and Gene Expression Between ALV-J-Positive and ALV-J-Negative Chickens
Source: Front Vet Sci. 2021 May 31;8:659840. doi: 10.3389/fvets.2021.659840 (PMC8203102; doi:10.3389/fvets.2021.659840)

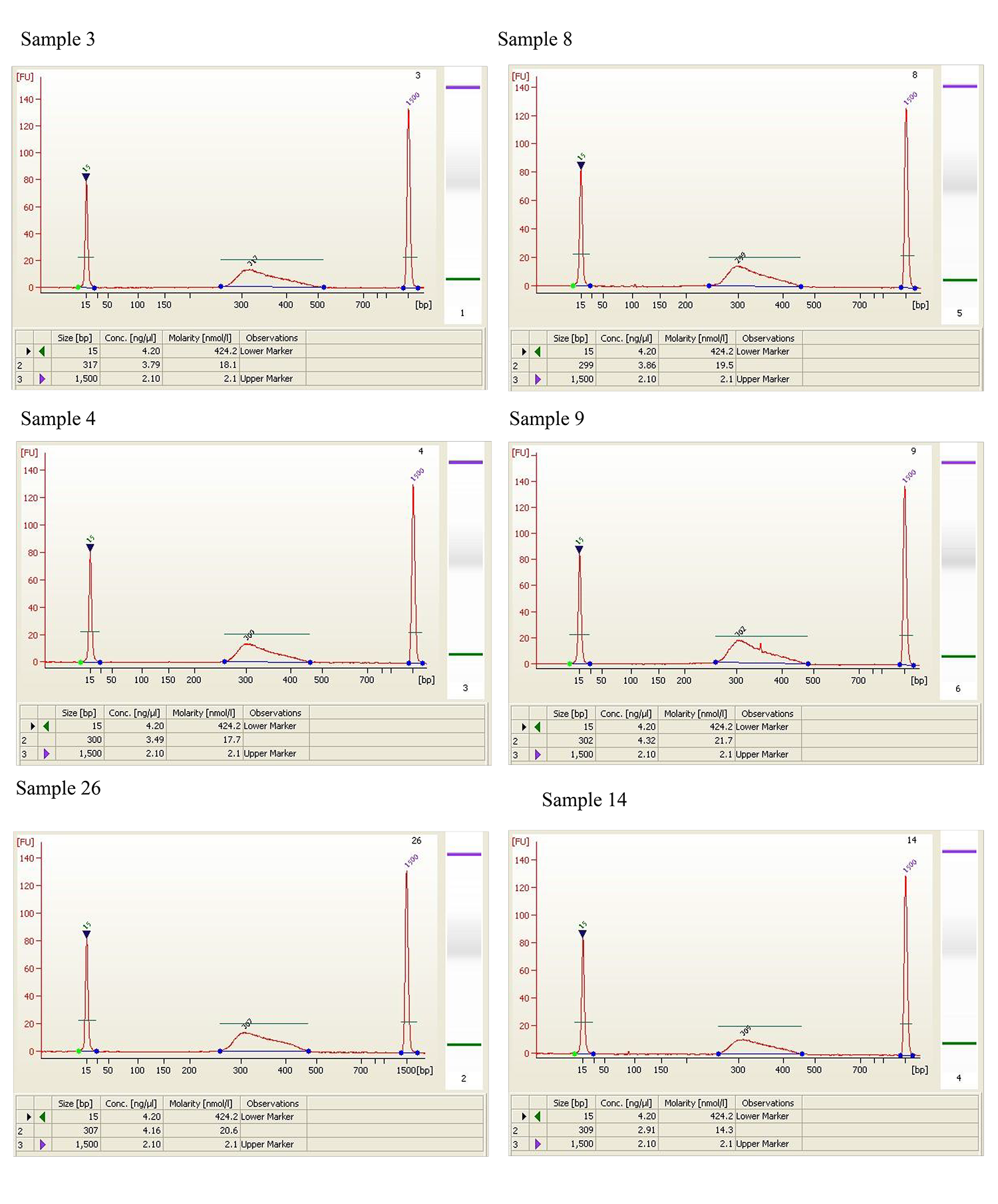

Supplement: Supplementary Figure 1 — Quality assessment of RNA-Seq library. [file Image_1.TIF]

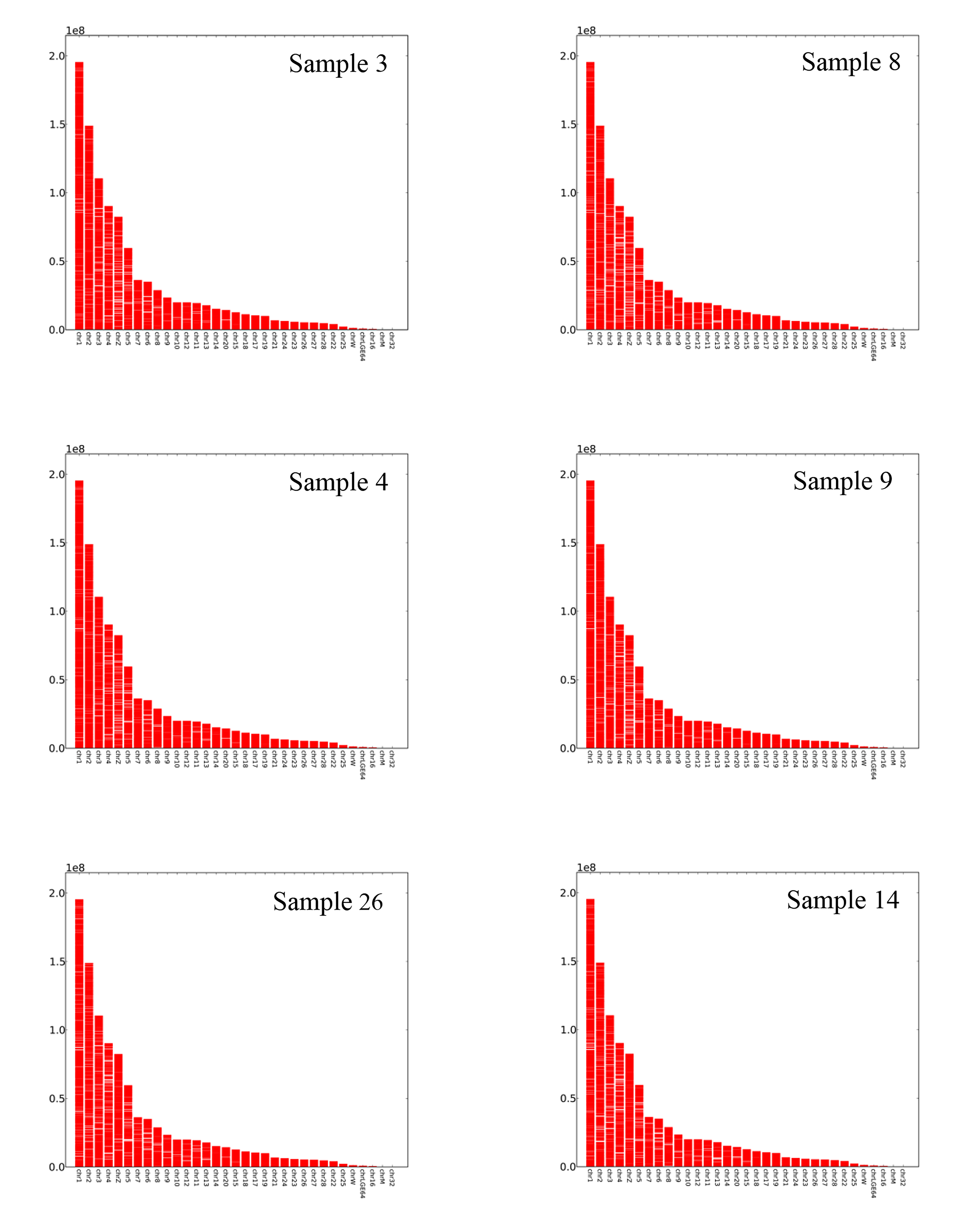

Supplement: Supplementary Figure 2 — Distribution of MeDIP-Seq reads on each chromosome in each sample. [file Image_2.TIF]
